# Evidence-Based and Emerging Dietary Approaches to Upper Disorders of Gut–Brain Interaction

**DOI:** 10.14309/ajg.0000000000001780

**Published:** 2022-04-13

**Authors:** Jan Tack, Hans Tornblom, Victoria Tan, Florencia Carbone

**Affiliations:** 1 Division of Gastroenterology and Hepatology, Leuven University Hospitals, Leuven, Belgium;; 2 Translational Research Center for Gastrointestinal Disorders (TARGID), Department of Chronic Diseases and Metabolism, University of Leuven, Leuven, Belgium;; 3 Rome Foundation for Disorders of Gut Brain Interactions (DGBIs), Chapel Hill, North Carolina, USA;; 4 Department of Molecular and Clinical Medicine, Institute of Medicine, Sahlgrenska Academy, University of Gothenburg, Gothenburg, Sweden;; 5 Department of Medicine, The University of Hong Kong, Hong Kong SAR.

## Abstract

Food ingestion is a major symptom trigger in functional esophageal and gastroduodenal disorders and gastroparesis. This review summarizes current knowledge and identifies areas of research on the role of food factors and the opportunities for dietary intervention in these disorders. While many patients experiencing functional esophageal and gastroduodenal disorders identify specific food items as symptom triggers, available data do not allow the identification of specific nutrient groups that are more likely to induce symptoms. In functional dyspepsia (FD), recent studies have shown the potential efficacy of a diet low in fermentable oligosaccharides, disaccharides, monosaccharides, and polyols, although the underlying mechanism of action is unclear. Reports of favorable responses to gluten elimination in patients with FD are confounded by the concomitant benefit of reduced intake of fructans, fermentable oligosaccharides, disaccharides, monosaccharides, and polyols present in wheat. Emerging data based on a 6-food elimination diet and confocal laser endomicroscopic evaluation of mucosal responses to food proteins suggest a role for duodenal allergic reactions in FD symptom generation. In patients with gastroparesis, a low-residue diet has been shown to improve symptoms. Novel dietary approaches under evaluation are the Mediterranean diet and the heating/cooling diet approach.

## INTRODUCTION

Dietary intervention studies for lower gastrointestinal tract disorders have led to diet advice being a well-established treatment option for irritable bowel syndrome (IBS) and other disorders of gut–brain interaction (DGBI) (1). The upper gastrointestinal tract is the port of entry for food and the site of first interaction between nutrients and the mucosa (2). Based on this consideration, a notable effect of dietary interventions would be expected for upper gastrointestinal DGBI also, but to date, this has been studied less intensively. This article summarizes the current knowledge on the role of nutrients in inducing symptoms in upper DGBI and the efficacy of dietary treatment interventions in these disorders.

## PHYSIOLOGY: UPPER GI FUNCTION RELATED TO FOOD INTAKE AND PROCESSING

Swallow-induced esophageal peristalsis propagates nutrient boluses aborally. Relaxation of the lower esophageal sphincter allows entry of the bolus into the stomach, with subsequent resumption of sphincter tone, which prevents gastroesophageal reflux (2). Transient lower esophageal sphincter relaxations (TLESR) are spontaneous relaxations of the lower esophageal sphincter and crural diaphragm, not triggered by swallowing, which allow venting of ingested air and gas but are also the main mechanism underlying postprandial gastroesophageal reflux events (3).

Between meals, the physiology of the upper gastrointestinal tract is characterized by the interdigestive migrating secretomotor complex, which is interrupted by ingestion of food, converting it to a fed-state physiology (2). Regarding food intake, the response of the upper gastrointestinal tract can be subdivided into 3 phases, depending on the level where nutrients are present. The cephalic phase is triggered by the sight, smell, or thought of food and initiates through the vagal efferents the secretion of saliva and gastric acid and pancreatic secretion and inhibition of upper gastrointestinal phasic motility and release of a number of peptide hormones such as gastrin and ghrelin (2). The gastric and intestinal phases require the presence and detection of food through a variety of sensory mechanisms.

A long-standing dogma states that the control of the gastric phase, when food resides in the stomach, is driven by volume sensing, whereas the intestinal phase, when food enters the duodenum, would also involve chemosensing (2,4). Gastric filling is associated with activation of tension-sensitive mechanoreceptors in the proximal stomach, which mediate the effect of intragastric pressure (IGP) on the occurrence of satiation (2,5). This is delayed by gastric accommodation, a relaxation of the proximal stomach, which is associated with a drop in IGP and increases volume tolerance (5).

The intestinal phase involves chemoreceptor activation by nutrients in the proximal small bowel, leading to release of several peptide hormones (including cholecystokinin [CCK], gastric inhibitory peptide, neurotensin, peptide YY, somatostatin, gastrin, secretin, and others) (2). Chemoreceptors involved included ion channels, transient receptor potential channels, and G protein–coupled tastant and nutrient-sensing receptors. At the same time, negative feedback vago-vagal reflexes, in synergy with hormonal effects, will inhibit gastric contractility and will slow down gastric emptying in response to the presence of nutrients, low pH, or hyperosmolar contents in the small intestine. Mechanical factors may also contribute to the control of the intestinal phase (2).

Besides its vital function to provide nutrients and energy, food intake is also associated with an important social and hedonic function, through the release of gut peptides and the activation of reward-related regions in the brain. The food reward system functioning may be altered in patients with DGBI (6).

## SEARCH STRATEGY

The search strategy is summarized in the Supplementary File (see Supplementary Digital Content 1, http://links.lww.com/AJG/C501).

## ESOPHAGEAL DISORDERS

### Role of food in symptom triggering

In patients with any type of dysphagia, deglutition of food is an obvious trigger (7). Typical symptoms of gastroesophageal reflux disease (GERD) mainly occur after meals when lower esophageal sphincter (LES) pressure is diminished and TLESR are triggered by gastric filling (3). Several studies have evaluated the role of food inducing GERD symptoms (8). Late evening meals are associated with increased time of supine acid exposure (8). In an Italian questionnaire study, 85% identified specific foods able to trigger GERD symptoms, especially spicy or fried foods, tomatoes, and chocolates (9). In a Korean survey, hot spicy stews, rice cakes, ramen noodles, fried foods, and topokki were the foods most frequently inducing symptoms (8). Spicy components and malic and citric acids present in tomatoes may underlie some of these experiences. Ingestion of a high-fat meal is associated with a decrease in LES pressure and increased reflux events, whereas a meal rich in proteins increased LES pressure (8,10,11). Ingestion of chocolates is associated with higher esophageal acid exposure (12). Alcohol intake causes a decrease in LES pressure and is associated with more reflux (8,13). In the prospective Nurses' Health Study that involves 48,308 women, intake of coffee, tea, or soda was associated with an increased risk of GERD symptoms (14), although an older meta-analysis failed to confirm a role for coffee intake (15). Carbonated drinks are also associated with an increased risk of GERD symptoms, and this may be due to their acidic pH and increased TLESR triggered by gastric distension (7,16). In a mechanistic study in 9 patients with GERD, chronic administration of fructo-oligosaccharides increased the number of TLESR, and this was associated with an increased secretion of glucagon-like peptide-1 (17). In healthy volunteers, acute administration of fructans, but not fructose, increased the number of TLESR in the fourth postprandial hour, and this was accompanied by increased flatulence, suggesting an effect through colonic fermentation (17).

### Food habits

Given the link between nutrient intake and esophageal symptoms, one would expect changes in dietary patterns in patients with disordered esophageal function. Surprisingly, little data are available on food habits in patients with chronic (functional) esophageal symptoms. In a preliminary report, total nutrient intake and macronutrient content did not differ significantly between patients with heartburn symptoms and healthy controls (18,19). There was also no difference in taking snacks in between meals, but patients with a higher reflux symptom severity skipped more meals (18,19). Similarly, it seems plausible that patients with functional dysphagia would adapt their food choices, going to aliments with softer consistency, which are easier to swallow. However, no data are available in the literature. In a preliminary report, patients with dysphagia without major abnormalities on high-resolution manometry showed no major differences in total caloric or macronutrient intake compared with controls (19). No correlations were found between dysphagia severity and nutrient intake and composition. In addition, the intake of dry foods was not altered in this dysphagia group. The same preliminary study also reported decreased intake of dietary fat in patients with globus (19).

### Dietary intervention: reflux avoiding and soft foods

Patients with GERD are often advised to refrain from fatty foods, late evening meals, coffee, alcohol, carbonated drinks, and orange juice (7). In an Italian study, elimination of perceived trigger foods in 100 patients with heartburn reduced symptom severity and eliminated heartburn or regurgitation symptoms in most of them (8). A preliminary report of a placebo-controlled crossover treatment trial in 8 patients with nonerosive heartburn showed increased heartburn symptoms after a 6-week intake of capsules with capsaicin, the active ingredient of red chili pepper and an agonist at transient receptor potential vanilloid 1 (TRPV1) channels (20), but a full study report has not been published to date. In a randomized open-label trial of a low fermentable oligosaccharide, disaccharide, monosaccharide, and polyol (FODMAP) diet versus usual dietary advice in 31 patients with symptomatic proton pump inhibitor-refractory GERD symptoms, no difference in control of GERD and dyspeptic symptoms was observed (21). In 38 GERD patients from Italy, an *in vitro* leukocyte reaction test (leukocytotoxic test) to food was used as a basis for randomization to a true or sham elimination diet for 4 weeks, which led to a lower GERD impact scale in the true diet group (22). No follow-up intervention studies have been published.

In patients with functional dysphagia, careful chewing and diet adaptations, ranging from avoidance of hard foods to semisolid, mixed, and liquid meals is advocated (7). The literature lacks case series and follow-up data documenting the efficacy of such interventions.

## FUNCTIONAL DYSPEPSIA AND GASTROPARESIS

### Role of food in symptom triggering

The biggest subgroup in functional dyspepsia (FD), postprandial distress syndrome (PDS), is characterized by symptoms of postprandial fullness and early satiation, which occur during or immediately after a meal, whereas the epigastric pain syndrome subtype lacks this immediate relation to meal intake (23,24).

An extensive meta-analysis investigating the reported relationships between food components and symptoms in patients with FD described no clear relationship between specific symptoms and different drinks (e.g., alcoholic beverages, carbonated drinks, milk, tea, and coffee) or foods (e.g., grain, pasta, wheat products, fruit, red bell pepper, and processed food) (25). Several studies identified fat as a key factor triggering symptoms, whereas carbohydrates seem less correlated with symptom induction (26–34).

Similar complexity was observed in patients with gastroparesis where fatty foods, foods containing capsaicin, and roughage-based foods were reported to commonly provoke symptoms, whereas other items such as ginger ale, gluten-free foods, tea, sweet potatoes, pretzels, white fish, clear soup, salmon, potatoes, white rice, popsicles, and applesauce could alleviate symptoms (35). Interestingly, the amount of carbohydrates in the alleviating or well-tolerated food seemed to be higher compared with those in the symptom-provoking food (35). Another study in 12 patients with gastroparesis showed that low-fat or liquid meals were better tolerated than high-fat, solid meals (36).

The general propensity of experiencing symptoms triggered by eating is a risk factor for decreased caloric intake, which is reflected in weight loss, estimated to occur in 55% of patients with FD (37). In patients with gastroparesis, whereas up to 10% of patients were reported to be underweight, a larger proportion was overweight (20%) or even obese (29%) (38). A diet that resulted in ≥5% increased body weight in patients with idiopathic gastroparesis led to improved symptoms, suggesting that body weight is an important determinant of gastroparesis symptoms (38).

The pathophysiology of food triggering symptoms in patients with FD and gastroparesis is incompletely understood. Most studies are without any clear association between gastric emptying time and overall symptom severity (39,40). Soluble fibers, lipids, and carbohydrates were all shown to decrease gastric emptying rate and to be associated with upper gastrointestinal symptoms (41–45). It has been suggested that in a proportion of these patients, an abnormal intestinal feedback of nutrients may result in a more intense inhibition of gastric emptying and enhanced gastric mechanosensitivity (46–49). Foods rich in fiber and fat, especially long-chain triglycerides, trigger the release of CCK, which reduces gastric motility and sensitizes the stomach to distention, which may be exaggerated in FD (33,49). Furthermore, it has been shown that FD symptoms induced by a fatty meal or intraduodenal lipid infusion were improved by lipase supplementation or a CCK-A receptor antagonist (50,51).

In up to 40% of patients with FD or gastroparesis, abnormal gastric accommodation to a meal with redistribution of the content toward the antrum is present and associated with symptoms of early satiation and weight loss (37,52,53). Moreover, duodenal nutrient exposure has been shown to contribute to the size of the gastric accommodation reflex and hence meal-induced satiation (54). Recently, PDS symptoms, such as early satiation, have been associated with increased duodenal eosinophil and mast cell numbers in patients with FD, and this is correlated to increased duodenal mucosal permeability (55–57). Early evidence suggests a role for nutrients triggering these events because preliminary reports show improvement of duodenal barrier function after 6 weeks of a low FODMAP diet in patients with FD (58), and exposure to proteins commonly associated with food allergy are able to acutely induce increased permeability (59,60).

### Food habits

In both FD and gastroparesis, patients are not able to tolerate large amounts of food and therefore tend to decrease the number of meals and calories and increase the number of snacks, with a risk of deficiency in calories, vitamins, and minerals (25–32,61–63). Available studies show that up to 80% of patients with FD report food avoidance (23), but findings on altered macronutrient intake are inconsistent with reports of lower intake of lipids, fibers, or carbohydrates (24–28).

The patient awareness of symptoms being triggered by meal intake might contribute to psychological distress. Indeed, stress and anxiety are more associated with PDS than epigastric pain syndrome (64). This could increase the risk to develop avoidant/restrictive food intake disorder (ARFID) that can further complicate their gastrointestinal symptoms. A study where patients with gastroparesis and FD completed self-report surveys for gastrointestinal symptom severity and symptoms of feeding/eating disorders showed that up to 40% reported significant ARFID according to the questionnaire (65). In patients with gastroparesis, the level of ARFID was more related to the severity of symptoms than to the gastric emptying rate, indicating the need to consider the presence of ARFID in dietary management of patients with gastroparesis and probably also FD, although this requires additional studies (65).

## DIETARY INTERVENTIONS

### Low FODMAP diet

**Figure 1.**
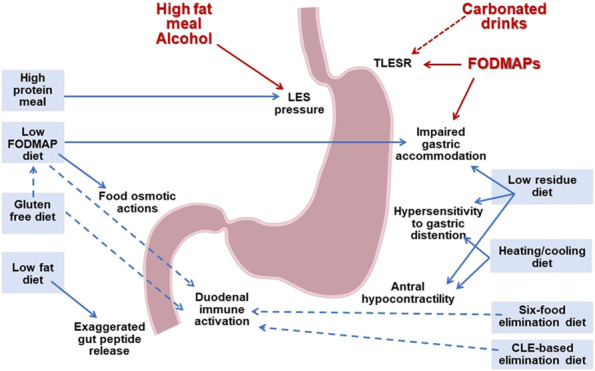
Schematic overview of pathogenetic mechanisms in functional dyspepsia and gastroparesis and the putative effects of therapeutic dietary interventions. CLE, confocal laser endomicroscopy; FODMAP, fermentable oligosaccharides, disaccharides, monosaccharides, and polyols; LES, lower esophageal sphincter; TLESR, transient lower esophageal sphincter relaxation.

Restricting the intake of poorly absorbed or undigested short-chain carbohydrates called FODMAP was shown to alleviate abdominal symptoms in IBS (66). In FD, a small number of recently published studies have investigated the therapeutic efficacy of this diet (Table 1 and Figure 1). A beneficial effect of a low FODMAP diet compared with standard dietary advice was reported in a cohort of 59 patients with FD, but with 81% having coexisting IBS (67). Goyal et al. (68) compared the effect of a 4-week low FODMAP diet to traditional dietary advice in a group of 105 patients with Rome IV FD where those with overlapping IBS and other dietary-related conditions (e.g., a history of lactose/fructose intolerance and patients already on restricted diet) were excluded. Symptoms improved in 67% after a low FODMAP diet compared with 57% after traditional dietary advice, mainly in the PDS subgroup and without significant difference between diets. Furthermore, durable benefit at 12 weeks was present in 46% and 41%, respectively (68). In a pilot study, the effect of a low FODMAP and gluten-free diet was explored in 11 patients with FD, of whom only 9 completed the elimination phase. A low FODMAP diet was associated with a nonsignificant trend toward overall symptom improvement (69). A preliminary report on 25 patients with FD showed a clinically significant symptom improvement in 62% after 6 weeks of a low FODMAP diet (58). Improvement occurred both in overall and individual symptom scores of postprandial fullness, early satiation, and upper abdominal bloating. After 6 weeks, patients entered a blinded reintroduction phase where individual FODMAP were reintroduced for 1 week in the form of powders, with 2 days recovery in between and glucose as a control. The blinded reintroduction phase of the study showed that a wide variety of FODMAP induced symptom recurrence, which occurred in an individualized pattern. Mannitol and galacto-oligosaccharides were the FODMAP that most commonly triggered FD symptom recurrence (both 29%), followed by fructans (21%), sorbitol (14%), fructose (14%), and lactose (12%) (58).

**Table 1.**
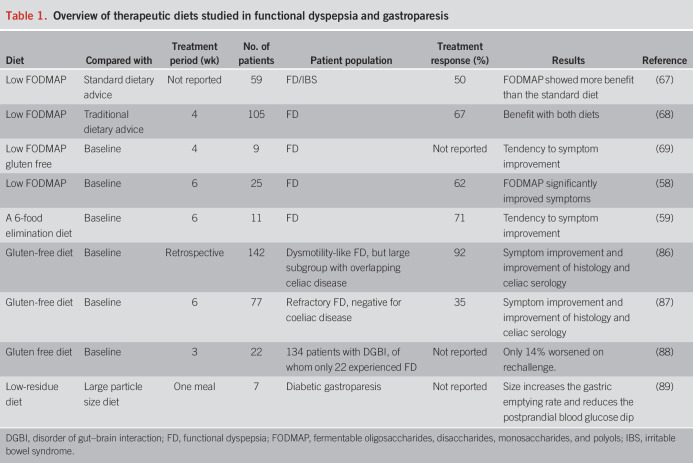
Overview of therapeutic diets studied in functional dyspepsia and gastroparesis

The mechanism underlying symptom reduction with dietary FODMAP restriction in patients with FD is unclear. In healthy controls and in patients with IBS, intragastric infusion of fructans, but not fructose or glucose, induced an early increase in IGP. This was associated with increased symptoms in IBS, suggesting that mechanisms other than osmotic or fermentation effects in the bowel may contribute to symptom generation (70). In a small pilot study, no association was found between baseline eosinophil counts and changes in dyspeptic symptoms during a low FODMAP diet (69). However, there are indications that FODMAP may affect duodenal mucosal integrity, as in the study by Van den Houte et al. (58), where symptom improvement during a low FODMAP diet correlated with increased transepithelial electrical resistance across the duodenal mucosa. Based on the emerging evidence, a low FODMAP diet can be considered in FD. However, future research will be needed to determine whether this dietary approach can be identical to the one in IBS or would benefit from FD-specific adaptations. Moreover, the place of the diet in the management algorithm relative to pharmacological therapies needs to be determined.

### Heating/cooling diet

Chinese nutrition therapy has been pervasively practiced in East Asia for more than 1000 years and was used by more than 50% of study subjects in a Hong Kong cohort (74). It stems from a holistic view of the body and mind, with the body optimally being in state of balance regarding yin (cooling) and yang (heat) (71). It is believed that imbalance in the body can be corrected by the ingestion of food, which has innate properties when interacting with the body (71–73). Excessive heat in the body is associated with symptoms of constipation, and excessive cooling in the body is associated with diarrhea, dyspepsia, belching, and excessive gaseous symptoms. The foods believed to generate heating (e.g., ginger, chili, and mutton) or cooling (e.g., bitter melon, tomatoes, and pears) in the body vary between regions of the far east and seem to be evolving slightly over time.

A small randomized controlled study found that ginger, commonly prescribed to reduce excess cold in the body, accelerated gastric emptying and increased antral contractions, as measured by ultrasonography, but without changes in dyspeptic symptoms or gastrointestinal peptide profiles (75). Interestingly, a combination of ginger and artichoke extract did improve dyspeptic symptoms (76). Chili, another commonly consumed condiment used to reduce cooling in the body and increase heat (77), was shown to decrease dyspeptic and GERD symptoms when chronically ingested (78). It should be noted that the heating and cooling concept as the basis of this diet is not based on physiological understanding and concepts. Some of the food components implicated in the heating/cooling diet have the potential to exert physiological effects through their actions on TRP channels, for instance, for chili and ginger through TRPV1 and TRPA1 channels (2). Based on the current data, this diet cannot be recommended for patients with FD.

### Confocal laser endomicroscopy–based diet and 6-food elimination diet

There is emerging evidence for a role of proteins as triggers of symptoms in FD. A large population-based study has shown a link between food allergies and FD (79), and another study showed higher IgG antibody titers to egg and soybean in patients with FD compared with those in controls and lower IgG antibody titers to crab, egg, shrimp, and wheat in patients with FD compared with those in patients with IBS (80). Initial reports from probe-based confocal laser endomicroscopy in patients with IBS and no immunoglobulin E antibodies to food revealed that application of food proteins on the duodenal mucosa can trigger acute disruption of the epithelial barrier integrity (81,82). These data were recently repeated in patients with FD/PDS also, showing a significant symptomatic response in 71% of patients with FD/PDS to a 6-food elimination diet and reporting confocal laser endomicroscopy findings of food-triggered disruption of the epithelial barrier in parallel with loss of mucosal integrity, as measured on biopsies mounted in Ussing chambers (59,60) (Table 1 and Figure 1). For this reason, identification and elimination of the specific food protein antigen(s) causing mucosal reactions may offer a potentially attractive management option for FD by preventing immune activation and disruption of the mucosa (82). However, implementation of the 6-food elimination diet for FD in clinical practice awaits larger clinical trials and experience.

### Gluten-free diet

Nonceliac gluten or wheat sensitivity is characterized by self-reported adverse symptoms of wheat sensitivity in the absence of demonstrable wheat allergy or celiac disease (83). Symptoms overlap with IBS, but more than half of those with nonceliac wheat sensitivity experience dyspeptic symptoms with reports of duodenal eosinophilia, which raises the possibility that wheat or gluten may be responsible for the genesis of symptoms in a subset of patients with FD (84,85). Symptom improvement after withdrawal of gluten has been reported in patients with FD (Table 1 and Figure 1). A retrospective study suggested that more than 90% of patients with nonulcer dyspepsia with duodenal Marsh-type lesions responded to a gluten-free diet and improved abnormal duodenal pathology or celiac serology (86). However, these patients may experience celiac disease rather than FD. The effect of wheat in patients with FD may be mediated by the fructan content, as discussed in the low FODMAP diet, through the effect of gluten itself (atypical allergy) or by other wheat components, including amylase trypsin inhibitors, which are nongluten proteins capable of activating the innate immune system. In a randomized crossover study that included 77 patients with refractory FD, only 35% (n = 27) experienced benefit after 6 weeks on a gluten-free diet, and only 5 deteriorated after a blinded gluten challenge, suggesting the benefit of a gluten-free diet mediated by factors other than gluten (87). In a pilot trial in 11 patients with FD on a low FODMAP, low gluten diet, there was a nonsignificant tendency for symptom improvement, but this study was underpowered and could not differ between elimination effects of gluten or fructans (69). In another study including 134 patients with DGBI, of whom only 22 experienced FD, more than 70% of patients responded to a gluten-free diet, but there were no statistically significant differences between patients who were subsequently challenged with gluten or placebo, again suggesting that FODMAP rather than gluten may be the cause of symptoms (88). Taken together, the evidence for a gluten-free diet in FD is limited and suggests that the symptom-reducing effects in FD can be due to gluten itself, reduced FODMAP content (most likely), or other mechanisms. The gluten-free diet cannot be recommended for clinical practice at present.

### Low-residue diet

The effects of the physical properties of a meal have only been formally assessed in patients with gastroparesis. In diabetic gastroparesis, meals with small particle size improve gastric emptying rate compared with large particle size meals (89), and this was associated with reduced gastroparesis symptoms, including associated symptoms of gastroesophageal reflux (90) (Table 1 and Figure 1). A small crossover study in patients with diabetic and idiopathic gastroparesis showed less intense and less frequent upper gastrointestinal symptoms after a low-fat vs high-fat solid meal and a further symptom reduction after both a high-fat and low-fat liquid meal, with the least symptoms after the latter (36). Small particle size or liquid nutrition can be recommended in gastroparesis based on these findings, but the relative efficacy of the diet versus pharmacological treatment approaches requires additional studies.

### Mediterranean diet

The Mediterranean diet comprises food and eating habits of countries bordering the Mediterranean sea (e.g., Italy, Spain, France, and Greece). It includes a high intake of vegetables, fruits, legumes, cereals, grains, fish, and unsaturated fats such as olive oil and reduced intake of meat and dairy foods (91). The Mediterranean diet has been associated with a beneficial role in cardiovascular disease prevention and depression (92–94). Recent studies in adults and in children and adolescents showed that poor adherence to the Mediterranean diet is associated with symptoms of IBS and FD (95,96). The effect of implementing this diet in FD or gastroparesis has not been investigated. Theoretical potential benefits may relate to high antioxidant and polyphenol contents, exerting anti-inflammatory effects, and to increased diversity of the gut microbiome (97–99). On the contrary, the Mediterranean diet is rich in FODMAP. Given the lack of prospective interventional data, the diet cannot be recommended for clinical practice at present.

## CONCLUSION

Overall, the effect of diet as a trigger of upper DGBI symptoms has the potential to target both gastric sensorimotor function and duodenal mucosal alterations and gut peptide release. While there are emerging signs of efficacy of a low FODMAP, low-residue, gluten-free, or 6-food elimination diet, we are still at an early phase of understanding the intricate mechanistic interactions and the therapeutic benefits. Only a low FODMAP diet for FD and a low-residue diet for gastroparesis have sufficient (emerging) evidence for use in clinical practice. In esophageal disease, soft foods are used to manage dysphagia, and fat or residue restriction can help diminish heartburn. Ongoing and future studies will allow us to define with more certainty the effects of dietary interventions and their therapeutic role in the management of upper DGBI.

## CONFLICTS OF INTEREST

**Guarantor of the article:** Jan Tack, MD, PhD.

**Specific author contributions:** All authors: reviewing final document for content. J.T.: literature search, draft section writings, introduction, and section on esophageal disorders. F.C.: section of functional dyspepsia, FODMAP, and confocal laser endomicroscopy based. V.T.: section on gluten elimination and cold/hot diet. H.T.: section on gastroparesis.

**Financial support:** None to report.

**Potential competing interests:** None to report.

**Study Highlights**

**WHAT IS KNOWN**

✓ Food ingestion is a major symptom trigger in functional esophageal and gastroduodenal disorders and gastroparesis.
✓ This review summarizes current knowledge and identifies areas of research on the role of food factors and the opportunities for dietary intervention in these disorders.

**WHAT IS NEW HERE**

✓ In functional dyspepsia, recent studies show potential efficacy of a diet low in fermentable oligosaccharides, disaccharides, monosaccharides, and polyols.
✓ Reports of favorable responses to gluten elimination in functional dyspepsia are confounded by the concomitant benefit of reduced intake of fructans.
✓ Emerging data based on a 6-food elimination diet and confocal laser endomicroscopic evaluation of mucosal responses to food proteins suggest a role for duodenal allergic reactions in functional dyspepsia symptom generation.
✓ In gastroparesis, a low-residue diet has been shown to improve symptoms.

## Supplementary Material

SUPPLEMENTARY MATERIAL
