# Supplementary material for: Evidence-Based and Emerging Dietary Approaches to Upper Disorders of Gut–Brain Interaction
Source: Am J Gastroenterol. 2022 Apr 13;117(6):965–72. doi: 10.14309/ajg.0000000000001780 (PMC9169754; doi:10.14309/ajg.0000000000001780)
Supplement: SUPPLEMENTARY MATERIAL [file acg-117-965-s001.docx]

## Search strategy

**Role of diet in symptom triggering**

(("Dyspepsia"[Mesh] OR "Dyspepsia"[tiab] OR "Functional Dyspepsia"[tiab] OR "postprandial distress syndrome"[tiab] OR "epigastric pain syndrome"[tiab] OR "Gastroparesis"[Mesh] OR "Gastroparesis"[tiab] OR “GERD”[tiab] OR “gastroesophageal reflux”[tiab] OR “esophageal motility disorder”[tiab] or “esophageal dysmotility"[tiab])

*AND*

*("symptom trigger"[tiab] OR "epigastric symptom*"[tiab] OR "gastric symptom*"[tiab] OR "postprandial fullness"[tiab] OR "Satiation"[Mesh] OR "satiation"[tiab] OR "early satiation"[tiab] OR "Visceral Pain"[Mesh] OR "epigastric pain"[tiab] OR "epigastric burning"[tiab] OR "Nausea"[Mesh:NoExp] OR "nausea"[tiab] OR "bloating"[tiab] OR "Vomiting"[Mesh:NoExp] OR "Vomiting"[tiab])) AND ("Diet"[Mesh] OR "diet"[tiab] OR "Diet, Food, and Nutrition"[Mesh:NoExp] OR "Food"[Mesh:NoExp] OR "Eating"[Mesh:NoExp] OR "Food Intake"[tiab] OR "Nutrient Intake"[tiab])*

**Food habits**

*("Dyspepsia"[Mesh] OR "Dyspepsia"[tiab] OR "Functional Dyspepsia"[tiab] OR "postprandial distress syndrome"[tiab] OR "epigastric pain syndrome"[tiab] OR "Gastroparesis"[Mesh] OR "Gastroparesis"[tiab]* OR “GERD”[tiab] OR “gastroesophageal reflux”[tiab] OR “esophageal motility disorder”[tiab] or “esophageal dysmotility"[tiab]*) AND*

*("Feeding Behavior"[Mesh:NoExp] OR "Food Habit*"[tiab] OR "Eating Habit*"[tiab] OR "Dietary Habit*"[tiab] OR "Diet Habit*"[tiab] OR "Food Preferences"[Mesh] OR "Food Preferences"[tiab])*

**Dietary intervention: FODMAP**

*((("Dyspepsia"[Mesh] OR "Dyspepsia"[tiab] OR "Functional Dyspepsia"[tiab] OR "postprandial distress syndrome"[tiab] OR "epigastric pain syndrome"[tiab] OR "Gastroparesis"[Mesh] OR "Gastroparesis"[tiab]* OR “GERD”[tiab] OR “gastroesophageal reflux”[tiab] OR “esophageal motility disorder”[tiab] or “esophageal dysmotility"[tiab*])*

*AND*

*("symptom trigger"[tiab] OR "epigastric symptom*"[tiab] OR "gastric symptom*"[tiab] OR "postprandial fullness"[tiab] OR "Satiation"[Mesh] OR "satiation"[tiab] OR "early satiation"[tiab] OR "Visceral Pain"[Mesh] OR "epigastric pain"[tiab] OR "epigastric burning"[tiab] OR "Nausea"[Mesh:NoExp] OR "nausea"[tiab] OR "bloating"[tiab] OR "Vomiting"[Mesh:NoExp] OR "Vomiting"[tiab])) AND ("Diet"[Mesh] OR "diet"[tiab] OR "Diet, Food, and Nutrition"[Mesh:NoExp] OR "Food"[Mesh:NoExp] OR "Eating"[Mesh:NoExp] OR "Food Intake"[tiab] OR "Nutrient Intake"[tiab]))*

*AND*

*("Diet, Carbohydrate-Restricted"[Mesh] OR "Carbohydrate Restricted"[tiab] OR "Carbohydrate-Restricted"[tiab] OR "Low Carbohydrate"[tiab] OR "Low-Carbohydrate"[tiab] OR "Dietary Carbohydrates"[tiab] OR "FODMAP*"[tiab] OR "Low FODMAP"[tiab] OR "Sugars"[Mesh] OR "Sugars"[tiab] OR "Oligosaccharides"[Mesh] "Oligosaccharide*"[tiab] OR "Disaccharides"[Mesh] OR "Disaccharide*"[tiab] OR "Lactose"[Mesh] OR "Lactose"[tiab] OR "Monosaccharides"[Mesh] OR "Monosaccharide*"[tiab] OR "Fructose"[Mesh] OR "Fructose"[tiab])*

**Dietary intervention: 6FED**

*("Dyspepsia"[Mesh] OR "Dyspepsia"[tiab] OR "Functional Dyspepsia"[tiab] OR "postprandial distress syndrome"[tiab] OR "epigastric pain syndrome"[tiab] OR "Gastroparesis"[Mesh] OR "Gastroparesis"[tiab]* OR “GERD”[tiab] OR “gastroesophageal reflux”[tiab] OR “esophageal motility disorder”[tiab] or “esophageal dysmotility"[tiab*]) AND*

*("Food Hypersensitivity"[Mesh] OR "Food Allergy"[tiab] OR "six food elimination diet"[tiab] OR "6 food elimination diet"[tiab] OR "6 food elimination diet"[tiab]*

**Dietary intervention: CLE-based**

*("Dyspepsia"[Mesh] OR "Dyspepsia"[tiab] OR "Functional Dyspepsia"[tiab] OR "postprandial distress syndrome"[tiab] OR "epigastric pain syndrome"[tiab] OR "Gastroparesis"[Mesh] OR "Gastroparesis"[tiab]* OR “GERD”[tiab] OR “gastroesophageal reflux”[tiab] OR “esophageal motility disorder”[tiab] or “esophageal dysmotility"[tiab] *NOT ("Animals"[Mesh] NOT "Humans"[Mesh])) AND ("Microscopy, Confocal"[Mesh:NoExp]*

*OR "confocal laser endomicroscopy"[tiab] OR "Confocal Microscopy"[tiab] OR "CLE"[tiab] OR "probe-confocal laser endomicroscopy"[tiab] OR "food challenge"[tiab] OR "food antigens"[tiab]* OR “GERD”[tiab] OR “gastroesophageal reflux”[tiab] OR “esophageal motility disorder”[tiab] or “esophageal dysmotility"[tiab *“)*

**Dietary intervention: Mediterranean diet**

*(*"Dyspepsia"[Mesh] OR "Dyspepsia"[tiab] OR "Functional Dyspepsia"[tiab] OR "postprandial distress syndrome"[tiab] OR "epigastric pain syndrome"[tiab] OR "Gastroparesis"[Mesh] OR "Gastroparesis"[tiab] OR “GERD”[tiab] OR “gastroesophageal reflux”[tiab] OR “esophageal motility disorder”[tiab] or “esophageal dysmotility"[tiab] OR *"Colonic Diseases, Functional"[Mesh:NoExp] OR "Irritable Bowel Syndrome"[Mesh] OR "Irritable Bowel"[tiab] OR "IBS" [tiab] OR "irritable colon"[tiab] OR "spastic colon"[tiab] NOT ("Animals"[Mesh] NOT "Humans"[Mesh]) OR review [pt] OR editorial [pt]) AND ("Mediterranean Diet"[tiab])*
